# Culture conditions and nutrition requirements for the mycelial growth of *Isaria farinosa* (Hypocreales: Cordycipitaceae) and the altitude effect on its growth and metabolome

**DOI:** 10.1038/s41598-018-33965-z

**Published:** 2018-10-23

**Authors:** Fei Liu, Meichun Xiang, Yanlei Guo, Xiaoli Wu, Guangxin Lu, Yong Yang, Xingzhong Liu, Shijiang Chen, Guozhen Zhang, Wangpeng Shi

**Affiliations:** 1 College of Plant Protection of China Agricultural University, Beijing, 100193 China; 2 Chongqing Academy of Chinese Materia Medica, Chongqing, 400065 China; 3 State Key Laboratory of Mycology, Institute of Microbiology, Chinese Academy of Science, Beijing, 100101 China; 4 College of Agriculture and Animal Husbandry, Qinghai University, Xining, 810016 China

## Abstract

*Isaria farinosa* is a pathogen of alpine *Thitarodes* larvae that are hosts for the Chinese medicinal fungus, *Ophiocordyceps sinensis*. A matrix analysis indicated that the optimal culture conditions for the mycelial growth of *I*. *farinosa* are a 50-mL liquid broth in a 250-mL flask at more than 100-rpm rotation and 15–25 °C. Illumination does not affect the mycelial growth. The optimal nutrition requirements are D-(+)-galactose and D-(−)-fructose as carbon resources and D-cysteine as well as yeast powder, peptone, and beef extract as nitrogen resources at a carbon-to-nitrogen ratio of 1:1 to 1:7. The mineral component and vitamins also significantly increase the mycelial growth of *I*. *farinosa*. Based on the optimal culture conditions and nutrition requirements for the mycelial growth of *I*. *farinosa*, the effects of altitude on mycelial growth and its metabolome were evaluated using quadrupole-time-of-flight/mass spectrometry, principal component analysis and partial least squares discriminant analysis. The altitude did not affect the mycelial production but significantly regulated its metabolome. The study presents a new approach to better select a method for producing more useful metabolites and highlights the necessity of establishing standards for culturing methods related to altitude to preserve fungal quality; additionally, the results indicate that the use of a fermenter may meet the demands of large-scale mycelial production.

## Introduction

*Isaria farinosa* (Holmsk.) Fr. 1832 [formerly *Paecilomyces farinosus* (Holmsk.) A.H.S. Br. & G. Sm. 1957] has been an important fungus in agriculture and pharmacy, and many studies have been conducted to identify the biological characteristics of the fungus; these studies have included collecting high-quality samples, finding the suitable temperature and moisture for its mycelial and spore growth, determining the range of its host, and additional topics^1^. Once the disease in *Thitarodes* (formerly *Hepialus*) larvae (Lepidoptera: Hepialidae), the host of *Ophiocordyceps sinensis* (Berk.) G.H. Sung, J.M. Sung, Hywel-Jones & Spatafora 2007, was found to be caused by *I*. *farinosa*^2^, the study of *I*. *farinosa* intensified, including not only its virulence characteristics^3,4^, fungicide^5,6^, biochemical characterisation^7^, biotransformation activity^8^ and molecular biology^9,10^ but also its secondary metabolites and pharmacological activity^11^.

The secondary metabolites extracted from *I*. *farinosa* have included alkaloids^12^, polysaccharides^13^, ketones^14^, quinones^15^ and more than 20 compounds^4^. Some of them have shown many pharmacological activities. For example, the paecilosetin extracted from *I*. *farinosa* can inhibit cellular proliferation of leukaemia^16^; Militarinones B isolated from *I*. *farinosa* was active against *Staphylococcus aureus* Rosenbach 1884, *Candida albicans* (C.P. Robin) Berkhout 1923 and *Streptococcus pneumoniae* (Klein) Chester 1884^17^; Jiang *et al*.^18^ reported that the intra- and extracellular polysaccharides from *I*. *farinosa* had the same antioxidant efficiency. In addition to the above, some chemical compositions from *I*. *farinosa* have shown hypoglycaemic action^19^ and antineoplastic activity^14,18,20^. Therefore, *I*. *farinosa* has the potential to be developed for medicinal purposes on a large scale.

There have been some studies on the factors that affect the mycelial growth of *I*. *farinosa*, such as the carbon and nitrogen sources and the optimal medium and temperature^6,21–23^. However, most previous studies were either aimed at biological control or were partial studies, and there have been no studies on systematically screening the suitable nutrition and culture conditions for mycelial growth from the perspective of preserving medicinal quality. Additionally, because the disease in *Thitarodes* larvae caused by *I*. *farinosa* develops differently at different altitudes during the practical production of artificially culturing Chinese *Cordyceps*, and because the strain used in the present study was obtained from *Thitarodes* larvae from the Tibetan Plateau, it was inferred that mycelial growth and its metabolome might be affected by altitude. Although the strains used in the studies^6,24^ were obtained from high altitude, the purpose of their works were biological control or increasing the survival rate of the larvae, and there have been no experiments about the effect of altitude on the mycelia and metabolome.

Therefore, based on these unexplored research questions and the previous studies, the effect of altitude on the mycelial growth and metabolome of *I*. *farinosa* were investigated after optimising the nutritional and cultured conditions used for mycelial growth. We hope that the results of this study provide parameters for more effectively utilising the fungus.

## Results

### Capacity bottling

The results showed that the mycelial weight was the highest in the treatment with the 50-mL capacity in the 250-mL Erlenmeyer flask (EF), and the weight was significantly different from the weights recorded in the other treatments (all *P* values were 0.00). The mycelial weight was the lowest in the treatment with a 200-mL capacity in the 250-mL EF, and the weight was significantly different from the weights recorded in the other treatments (all *P* values were 0.00). In the 50–200 mL capacity range for the 250-mL EFs, higher capacities led to lower mycelial weights. There was no significant difference between the mycelial weights in the treatment with 100-mL capacity in the 500-mL EF and the treatment with 100-mL capacity in the 250-mL EF (*P* = 0.87) (Table 1).

**Table 1:** Effect of bottling capacity on the mycelial growth of *Isaria farinosa*.

Bottling capacity (mL)	Mycelial dry weight (g L^−1^)*
50/250 mL	7.28 ± 0.28 a
100/250 mL	5.96 ± 0.14 b
150/250 mL	5.59 ± 0.37 b
200/250 mL	3.48 ± 0.71 c
100/500 mL	5.90 ± 0.24 b

*Mean ± SE. Values followed by the same letters within a column do not differ significantly (ANOVA, *P* = 0.05).

### Inoculum concentration

The results indicated that higher inoculum concentrations led to higher mycelial weights (Table 2). The mycelial weight was the highest in the treatment with the 20% inoculum concentration and lowest in the treatment with the 2% inoculum concentration. The one-way analysis of variance (ANOVA) results indicated that there was no significant difference among mycelial weights for the treatments with inoculum concentrations of 2, 5, 10 and 15% (*P*_2–5%_ = 0.49; *P*_2–10%_ = 0.09; *P*_2–15%_ = 0.17; *P*_5–10%_ = 0.28; *P*_5–15%_ = 0.48; *P*_10–15%_ = 0.70). The difference in the mycelial weight from the inoculum concentration of 20% was not significantly different from the treatments with the inoculum concentrations of 15% (*P* = 0.08) and 10% (*P* = 0.15).

**Table 2:** Effect of inoculum concentration on the mycelial growth of *Isaria farinosa*.

Inoculum concentration (%, v v^−1^)	Mycelial dry weight (g L^−1^)*
2	6.25 ± 0.46 b
5	6.90 ± 0.70 b
10	7.91 ± 2.06 ab
15	7.55 ± 0.37 ab
20	9.28 ± 0.92 a

*Mean ± SE. Values followed by the same letters within a column do not differ significantly (ANOVA, *P* = 0.05).

### Rotation speed

It was clear that the mycelial weight was the lowest in the treatment with a rotation speed of 0 rpm; in contrast, the weight was the highest in the treatment with a rotation speed of 120 rpm (Table 3). The ANOVA results indicated that the difference in the mycelial weight in the treatment with a rotation speed of 0 rpm was significantly different compared with the mycelial weights in the treatments with other rotation speeds (all *P* values were 0.00). However, there were no significant differences among the mycelial weights in the treatments with any rotation speed above 0 in the present experiment (*P*_100–120 rpm_ = 0.50; *P*_100–150 rpm_ = 1.00; *P*_100–180 rpm_ = 0.69; *P*_120–150 rpm_ = 0.50; *P*_120–180 rpm_ = 0.30; *P*_150–180 rpm_ = 0.70).

**Table 3:** Effect of rotation speed on the mycelial growth of *Isaria farinosa*.

Rotation speed (rpm)	Mycelial dry weight (g L^−1^)*
0	2.14 ± 0.54 b
100	7.33 ± 2.22 a
120	7.95 ± 2.22 a
150	7.32 ± 0.47 a
180	6.97 ± 0.58 a

*Mean ± SE. Values followed by the same letters within a column do not differ significantly (ANOVA, *P* = 0.05).

### Illumination

The research indicated that the mycelial weight was the highest in the treatment with 24 h of darkness, and the weight was the lowest in the treatment with 24 h of light. The ANOVA results indicated there were no significant differences among the mycelial weights of the three treatments (*P*_12 h of light/12 h of darkness−24 h of light_ = 0.54; *P*_12 h of light/12 h of darkness−24 h of darkness_ = 0.90; *P*_24 h of darkness−24 h of light_ = 0.46) (Table 4).

**Table 4:** Effect of illumination on the mycelial growth of *Isaria farinosa*.

Illumination	Mycelial dry weight (g L^−1^)*
12 h of light/12 h of darkness	5.96 ± 0.28 a
24 h of light	5.74 ± 0.49 a
24 h of darkness	6.00 ± 0.41 a

*Mean ± SE. Values followed by the same letters within a column do not differ significantly (ANOVA, *P* = 0.05).

### Temperature

The growth rate of the mycelia at 20 °C was the largest after 5 d, followed by 25 °C, and the mycelia did not grow at 0 and 35 °C (Table 5). The differences in growth rates among the 15, 20 and 25 °C conditions were not significant (*P*_15–20 °C_ = 0.08; *P*_15–25 °C_ = 0.25; *P*_20–25 °C_ = 0.54), and all them were significantly different from the growth rates of the mycelia at 5, 10 and 30 °C (Except *P*_15–10 °C_ = 0.10, *P*_15–30 °C_ = 0.08 and *P*_15–5 °C_ = 0.02, *P*_25–10 °C_ = 0.01, *P*_25–30 °C_ = 0.01, the other *P* values were 0.00). The result of verification showed that the differences in mycelial yield in liquid fermentation were not significant (*P*_20–15 °C_ = 0.66, *P*_20–25 °C_ = 0.40 and *P*_25–15 °C_ = 0.68); however, the mycelial dry weight at 20 °C was the highest, consistent with the results on agar medium (Table 5).

**Table 5:** Effect of temperature on the mycelial growth of *Isaria farinosa*.

Temperature (°C)	Growth rate 5 d later (mm/d)*	Mycelial dry weight 5 d later (g L^−1^)*
0	0 d	—
5	0.93 ± 0.08 b	—
10	1.28 ± 0.12 b	—
15	1.82 ± 0.16 a	5.87 ± 0.19 a
20	2.25 ± 0.18 a	5.92 ± 0.08 a
25	2.10 ± 0.78 a	5.83 ± 0.06 a
30	1.10 ± 0.1 b	—
35	0 c	—

*Mean ± SE. Values followed by the same letters within a column do not differ significantly (ANOVA, *P* = 0.05).

^—^No experiment to verify.

### Carbon source

Among the 16 tested carbon sources, the organic acid did not benefit the growth of the mycelia. The highest mycelial yield was obtained with D-(+)-galactose, followed by D-(−)-fructose. Very weak growth was observed in the medium containing citric acid and in the control medium (Table 6). However, the ANOVA results showed that the mycelial yield in the D-(+)-galactose medium was not significantly different from that in the media containing D-(−)-fructose (*P* = 0.97), soluble starch (*P* = 0.45), D-(+)-trehalose (*P* = 0.34), D-(+)-cellobiose (*P* = 0.25), sucrose (*P* = 0.18) and D-(+)-glucose (*P* = 0.05). Very weak growth was observed in the media with glucitol or citric acid as the carbon sources, and both mycelial yields treated with these carbon sources were significantly low (*P*_citric acid-glucitol_ = 0.75; *P*_citric acid-control_ = 0.97; *P*_glucitol-control_ = 0.79).

**Table 6:** Effect of carbon source on the mycelial growth of *Isaria farinosa*.

Carbon source		Mycelial dry weight (g L^−1^)*
Monosaccharides	D-(+)-Galactose	3.45 ± 0.41 a
	D-(−)-Fructose	3.43 ± 0.49 a
	D-(+)-Glucose	2.79 ± 0.11 abc
	D-(+)-Xylose	1.78 ± 0.40 d
	D-(+)-Trehalose	3.13 ± 0.23 ab
	Mannose	2.50 ± 0.96 bc
	D-(−)-Ribose	2.24 ± 0.22 cd
	L-(−)-Sorbose	1.74 ± 0.16 d
Disaccharides	D-(+)-Cellobiose	3.06 ± 0.27 ab
	Maltose	2.66 ± 0.09 bc
	Sucrose	2.99 ± 0.59 abc
Polysaccharides	Soluble starch	3.19 ± 0.26 ab
	Cellulose	ND
Alditols	D-Mannitol	2.55 ± 0.65 bc
	Glucitol	0.20 ± 0.11 e
Control		0.11 ± 0.14 e
Organic acid	Citric acid	0.10 ± 0.17 e

*Mean ± SE. Values followed by the same letters within a column do not differ significantly (ANOVA, *P* = 0.05).

### Nitrogen source

Among the 19 nitrogen sources examined in this study, yeast powder was the most effective for increasing mycelial growth, followed by peptone. The mycelial yield was the lowest in the medium that lacked a carbon source or a nitrogen source (Table 7). The ANOVA results showed there were no significant differences among the mycelial yields in the treatments with yeast powder, peptone and beef extract (*P*_yeast powder-peptone_ = 0.82; *P*_beef extract-peptone_ = 0.30; *P*_beef extract-yeast powder_ = 0.20). The media supplemented with yeast powder, peptone or beef extract resulted in significantly higher mycelial yields than the yields that resulted from any other nitrogen sources (all *P* values were 0.00). Among all tested amino acids, L-cystine was the best nitrogen source for mycelial growth, followed by DL-glutamic acid. There were no significant differences among the mycelial yields for L-cystine, DL-glutamic acid, L-arginine, asparagine and DL-serine (*P*_L-cystine-DL-glutamic acid_ = 0.52; *P*_L-cystine-L-arginine_ = 0.39; *P*_L-cystine-asparagine_ = 0.14; *P*_L-cystine-DL-serine_ = 0.12; *P*_DL-glutamic acid-L-arginine_ = 0.82; *P*_DL-glutamic acid-asparagine_ = 0.39; *P*_DL-glutamic acid-DL-serine_ = 0.36; *P*_L-arginine-asparagine_ = 0.53; *P*_L-arginine-DL-serine_ = 0.49; *P*_asparagine-DL-serine_ = 0.96). The mycelial yields of glycine, L-histidine and L-aspartic acid were not significantly different from the yields of Control 1 and Control 2 (*P*_glycine-Control 1_ = 0.94; *P*_glycine-Control 2_ = 0.33; *P*_L-histidine-Control 1_ = 0.83; *P*_L-histidine-Control 2_ = 0.40; *P*_L-aspartic acid-Control 1_ = 0.82; *P*_L-aspartic acid-Control 2_ = 0.40).

**Table 7:** Effect of nitrogen source on the mycelial growth of *Isaria farinosa*.

Nitrogen source		Mycelial dry weight (g L^−1^)*
Inorganic nitrogen	Ammonium nitrate	0.90 ± 0.07 ef
	Sodium Nitrate	1.38 ± 0.66 def
	Urea	2.43 ± 0.49 bcd
Amino acids	L-Cysteine	3.20 ± 0.04 b
	DL-Glutamic acid	2.83 ± 0.51 bc
	L-Arginine	2.70 ± 0.82 bcd
	Asparagine	2.33 ± 0.18 bcd
	DL-Serine	2.30 ± 0.12 bcd
	L-Proline	1.78 ± 0.03 cde
	L-Phenylalanine	1.76 ± 0.42 cde
	DL-Threonine	1.69 ± 0.14 cde
	Methionine	1.60 ± 0.26 cde
	L-Lysine	1.41 ± 0.27 def
	Glycine	0.72 ± 0.05 ef
	L-Histidine	0.63 ± 0.07 ef
	L-Aspartic acid	0.63 ± 0.95 ef
Complex organic nitrogen	Yeast powder	6.23 ± 0.55 a
	Peptone	6.10 ± 2.72 a
	Beef extract	5.49 ± 0.38 a
Controls	Control 1	0.76 ± 0.36 ef
	Control 2	0.15 ± 0.08 f

*Mean ± SE. Values followed by the same letters within a column do not differ significantly (ANOVA, *P* = 0.05).

### Carbon-to-nitrogen (C/N) ratio

Among the eight C/N ratios, the mycelial yield for the treatment with a 1:1 ratio was the highest, followed by the treatment with the 7:1 ratio (Table 8). The ANOVA results showed there was no significant difference between the treatment with 1:1 and 7:1 ratio (*P* = 0.07), and both treatments were significantly different from the other treatments (all *P* values were 0.00). As the C/N ratio increased, the mycelial yield decreased, except for the observed increase in the treatment with the 56:1 ratio.

**Table 8:** Effect of C/N ratio on the mycelial growth of *Isaria farinosa*.

C/N ratio	D-(+)-glucose (g L^−1^)	C%^α^	beef extract (g L^−1^)	N%^β^	Total organic matter (g L^−1^)^γ^	Mycelial dry weight (g L^−1^)^δ^
1:1	3.25	11.13	8.45	11.13	11.70	4.08 ± 0.06 a
7:1	8.54	29.18	3.17	4.17	11.70	3.71 ± 0.21 a
14:1	9.87	33.74	1.83	2.41	11.70	3.22 ± 0.19 b
21:1	10.41	35.60	1.28	1.68	11.70	2.61 ± 0.12 c
28:1	10.71	36.61	0.99	1.31	11.70	2.02 ± 0.17 de
42:1	11.02	37.67	0.68	0.90	11.70	1.92 ± 0.35 de
56:1	11.18	38.23	0.52	0.68	11.70	2.15 ± 0.39 d
70:1	11.28	38.57	0.42	0.55	11.70	1.71 ± 0.10 e

^α^The carbon content of D-(+)-glucose was 40% based on the chemical formula of C_6_H_12_O_6_. C% equals the amount of D-(+)-glucose (g L^−1^) × 40%/total organic matter.

^β^The nitrogen content of the beef extract was determined as 15.41% by the Kjeldahl method. N% equals the amount of beef extract (g L^−1^) × 15.41%/total organic matter.

^γ^Total organic matter represents the total dry weight (g) of D-(+)-glucose and beef extract in 1 L of medium.

^δ^Mean ± SE. Values followed by the same letters within a column do not differ significantly (ANOVA, *P* = 0.05).

### Macro-element

Among all macro-element treatments, the mycelial yield was the highest in the complete medium without sodium, followed by the complete medium (Table 9). There were no significant differences among the yields of the treatments with Control 1, the complete medium without sodium, the complete medium without potassium and the complete medium without calcium (*P*_Control 1-the complete medium without sodium_ = 0.69; *P*_Control 1-the complete medium without potassium_ = 0.79; *P*_Control 1-the complete medium without calcium_ = 0.15; *P*_the complete medium without sodium-the complete medium without potassium_ = 0.50; *P*_the complete medium without sodium-the complete medium without calcium_ = 0.07; *P*_the complete medium without potassium-the complete medium without calcium_ = 0.23). The mycelial yield of Control 2 was the lowest, and it was significantly different from the yield of any other treatment (all *P* values were 0.00). Except for Control 2, the mycelial yield of the complete medium without magnesium was the lowest, but it was not significantly different from the complete medium without calcium (*P* = 0.08). It seems that magnesium is more indispensable than the other tested macro-elements.

**Table 9:** Effects of macro-element on the mycelial growth of *Isaria farinosa*.

Macro-element	Mycelial dry weight (g L^−1^)*
Control 1 (Complete medium)	2.92 ± 0.35 a
Control 2 (No macro-elements)	1.15 ± 0.16 c
Complete medium without potassium	2.86 ± 0.25 a
Complete medium without sodium	3.00 ± 0.26 a
Complete medium without magnesium	2.22 ± 0.05 b
Complete medium without calcium	2.60 ± 0.30 ab

*Mean ± SE. Values followed by the same letters within a column do not differ significantly (ANOVA, *P* = 0.05).

### Trace element

The mycelial yield was the highest among all of the trace element treatments when the medium lacked copper, followed by the complete medium (Table 10). There was no significant difference between the medium without copper and the complete medium (*P* = 0.08). However, the mycelial yield of the medium without copper was significantly different from the media without iron (*P* = 0.00), zinc (*P* = 0.00) and manganese (*P* = 0.00). The mycelial yield was the lowest in the medium without any trace elements, and it was lower than any other treatment. However, there was no significant difference between Control 2 and the lowest yield of mycelia in the complete medium without iron (*P* = 0.58). This result indicated that iron was more indispensable than the other trace elements tested.

**Table 10:** Effects of trace element on the mycelial growth of *Isaria farinosa*.

Trace element	Mycelial dry weight (g L^−1^)*
Control 1 (Complete medium)	1.56 ± 0.19 ab
Control 2 (No trace elements)	1.15 ± 0.16 c
Complete medium without copper	1.87 ± 0.38 a
Complete medium without iron	1.24 ± 0.14 bc
Complete medium without zinc	1.28 ± 0.10 bc
Complete medium without manganese	1.25 ± 0.06 bc

*Mean ± SE. Values followed by the same letters within a column do not differ significantly (ANOVA, *P* = 0.05).

Among the ten factors investigated above, from a practical perspective, the optimal nutritional requirements included D-(+)-glucose, beef extract, iron, magnesium, and a C/N ratio of 7:1; additionally, the optimal culture conditions included 50-mL bottling in a 250-mL EF with 10% inoculum, 120 rpm, 12 h light/12 h darkness, and 20 °C.

### Orthogonal test

According to the orthogonal matrix method and the magnitude of R (i.e., the maximum difference) (Table 11), among the three nutrition sources, the effect of beef extract had the greatest effect on mycelial growth (R = 1.20), followed by D-(+)-glucose (R = 0.07); in contrast, the effect of thiamine (V_B1_) (selected from the report^24^) on mycelial growth was the weakest (R = 0.05). The ANOVA results showed that beef extract had a significant effect on the mycelial growth of *I*. *farinosa*; meanwhile, the interactions of D-(+)-glucose and beef extract, D-(+)-glucose and V_B1_, and beef extract and V_B1_ had no significant effects on mycelial growth (Table 12 and Supplementary Tables S1–S3), which were consistent with the intuitive analyses (Table 11).

**Table 11:** L_8_(2^7^) orthogonal experiment.

Run	A	B	A × B	C	A × C	B × C	D	Mycelial weight (g L^−1^)*
1	1	1	1	1	1	1	1	3.84 ± 0.21
2	1	1	1	2	2	2	2	4.04 ± 0.49
3	1	2	2	1	1	2	2	5.04 ± 0.15
4	1	2	2	2	2	1	1	5.30 ± 0.17
5	2	1	2	1	2	1	2	4.16 ± 0.17
6	2	1	2	2	1	2	1	3.93 ± 0.86
7	2	2	1	1	2	2	1	5.23 ± 1.22
8	2	2	1	2	1	1	2	5.18 ± 0.35
K_1_	4.56	3.99	4.57	4.57	4.50	4.62	4.58	
K_2_	4.63	5.19	4.61	4.61	4.68	4.56	4.61	
R	0.07	1.20	0.04	0.05	0.19	0.06	0.03	

A, B, C, D are D-(+)-glucose, beef extract, V_B1_, and experimental error, respectively.

A × B, A × C and B × C represent the interactions between the factors D-(+)-glucose and beef extract, D-(+)-glucose and V_B1_, and beef extract and V_B1_, respectively.

*Values are the mean ± SE of triplicate determinations.

K_1_ and K_2_ are the mean value of level 1 and level 2, respectively.

R is the maximum of K_1_ and K_2_ minus the minimum of K_1_ and K_2_, respectively.

**Table 12:** Variance analysis of the L_8_(2^7^) orthogonal experiment on the optimisation of the culture medium.

Variance source	Sum of squares^α^	F-ratio	Significance level^β^
D-(+)-glucose	0.01	5.00	
Beef extract	2.86	1428.00	*^γ^
D-(+)-glucose × Beef extract	0.00	1.00	
V_B1_	0.00	2.00	
D-(+)-glucose × V_B1_	0.07	34.00	
Beef extract × V_B1_	0.01	3.50	
Experimental error	0.00		

^α^The degree of freedom in this experiment was 1, and the mean square of each factor was the same as the sum of squares.

^β^F0.05 (1, 1) = 161, F0.01 (1, 1) = 4052.

^γ^F-ratio > F0.05

Based on the significance of the effect of the three nutrition factors, the mycelial yield, the K value and the method, when the effect of the factor was significant, the mycelial yield and the K value were greater, and the nutritional composition was better for mycelial growth. Therefore, the optimum composition should be 4.00 g L^−1^ of beef extract, 1.50 mg L^−1^ of V_B1_ and 14.20 g L^−1^ of D-(+)-glucose. To confirm the ability of this medium to support high-yield growth, the mycelia were submerged in this optimised medium, and the output, 5.14 g L^−1^ of mycelial biomass, was similar to the 5.18 g L^−1^ obtained from the same medium tested in the orthogonal experiment. Therefore, the selected concentrations represented the ideal growth conditions.

### Effects of altitude on mycelial growth

After 5 d of fermentation, the treatment at an altitude of 430 m in Chongqing (29°31′54.26′′N, 106°35′55.50′′E) (low altitude, LA) produced 5.07 g L^−1^ mycelia; additionally, the treatment at an altitude of 2365 m in Xining in Qinghai Province (36°43′34.82′′N, 101°45′01.78′′E) (high altitude, HA) produced 5.10 g L^−1^ mycelia. The difference in the mycelial production between at the LA and the HA was not significant (*P* = 0.74).

### Non-targeted metabolome pattern analysis

The raw data of the study samples, namely, the total ion chromatograms, are presented in detail in the supplementary material (Supplementary Dataset S1). The collection time for these data was no longer than 10 min. After processing, more than 2300 features that yielded identical values for the mass-to-charge ratio (m/z) and retention time (RT) under specific conditions from all samples were exported into Excel spreadsheets.

**Figure 1:**
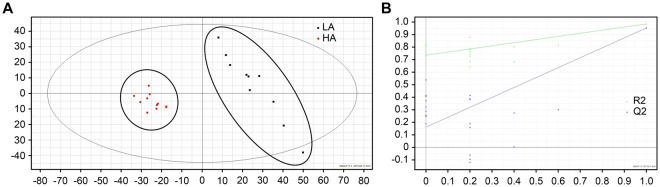
Score plots obtained from the non-targeted Q-TOF/MS analysis. (**A**) Qualitative profiling of the LA and the HA was conducted through PCA. Their metabolic patterns showed apparent separation. (**B**) Score plots of PLS-DA in the UPLC and positive ESI ion mode. Their metabolic patterns were fully discriminated from one another (R2Y = 0.98; Q2 = 0.95). The X-axis and Y-axis represent the two highest X-scores of dimensions 1 and 2 for matrix X of the mass ion abundances, respectively (black squares: LA; red circles: HA).

To investigate the metabolic patterns of the HA and LA, a principal component analysis (PCA) was first conducted on the datasets obtained under the analytical conditions, i.e., ultra-performance liquid chromatography (UPLC) and positive electrospray ionisation (ESI) ion mode. The score plots obtained from PCA were used for an unsupervised pattern analysis of all mycelial samples, and they are shown in Fig. 1. PCA showed apparent separation between the HA and LA in the UPLC and positive ESI ion mode (Fig. 1A). A supervised partial least squares discriminant analysis (PLS-DA) was applied to further indicate that the model does not exist without fitting (R2Y = 0.98; Q2 = 0.95) (Fig. 1B), which showed predictive ability to screen the differential variables between groups.

### Identification of differential metabolites

The quadrupole-time-of-flight/mass spectrometry (Q-TOF/MS) method provided identical molecular masses, RT and MS/MS spectra data for the candidates identified based on the variable importance in the projection (VIP) results obtained from the PLS-DA. Among the metabolites with a VIP value > 1.0 (Supplementary Dataset S2), 417 metabolites were downregulated, and 631 metabolites were upregulated in the LA compared with the HA. The 1048 compounds were further validated by Student’s t-test, and the features that showed significant differences (*P* < 0.05) (Supplementary Dataset S2) were identified and searched in the METLIN, the Mass Bank and the human metabolome database for further identification.

**Figure 2:**
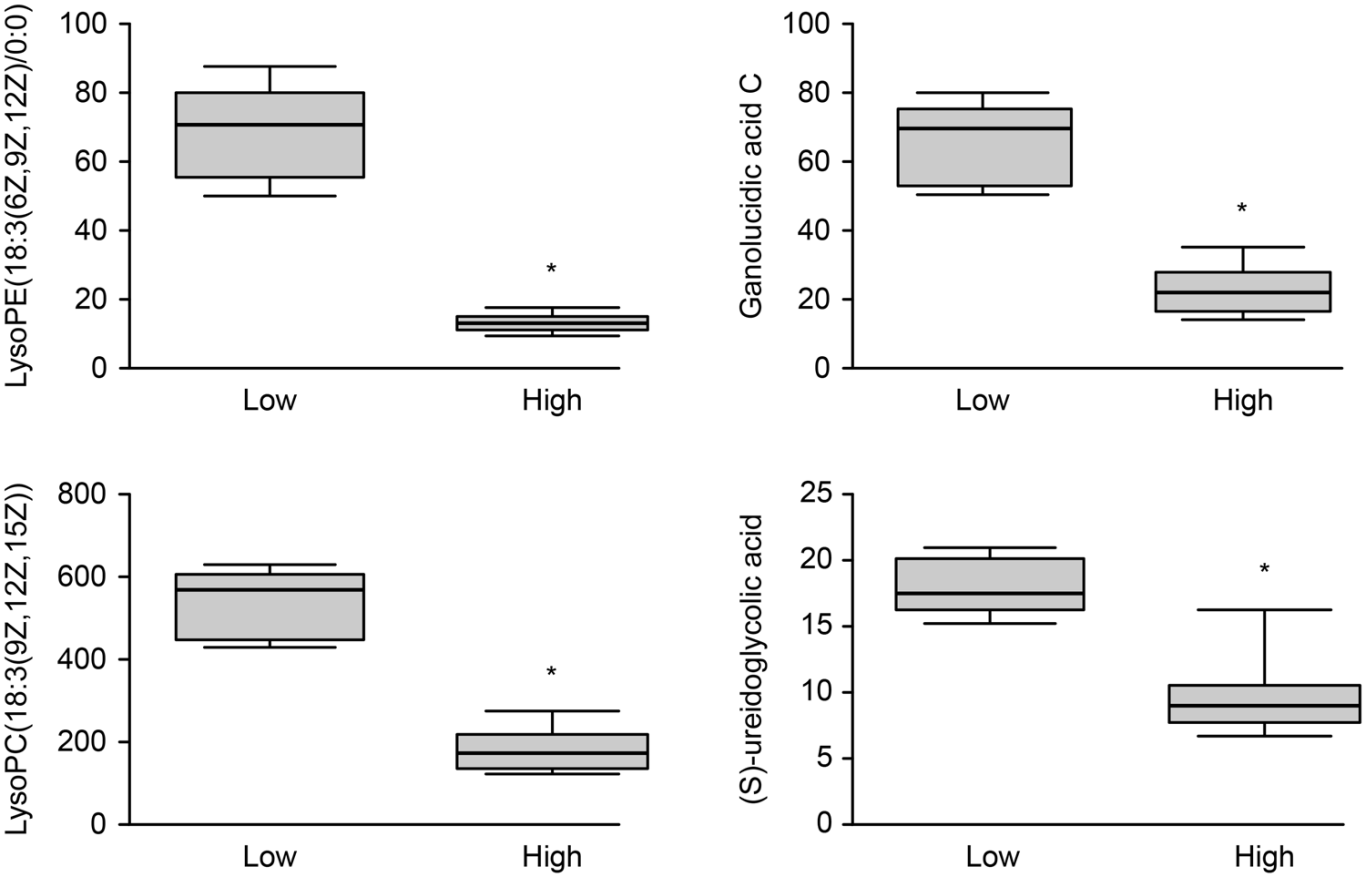
Metabolite profiles obtained from the quantitative analysis of the subjects. The figure was obtained using GraphPad Prism, and the names of metabolites are shown in the box plot. The box plot consists of the median (horizontal line) and the inter-quartile range, and the whiskers indicate the minimum and maximum values unless there were outliers, in which case the whiskers extend to a maximum of 1.5 times the inter-quartile range. The differences in LPC, ganolucidic acid C, LPE and (S)-ureidoglycolic acid between the LA and HA are displayed. “*” indicates *P* < 0.01.

**Figure 3:**
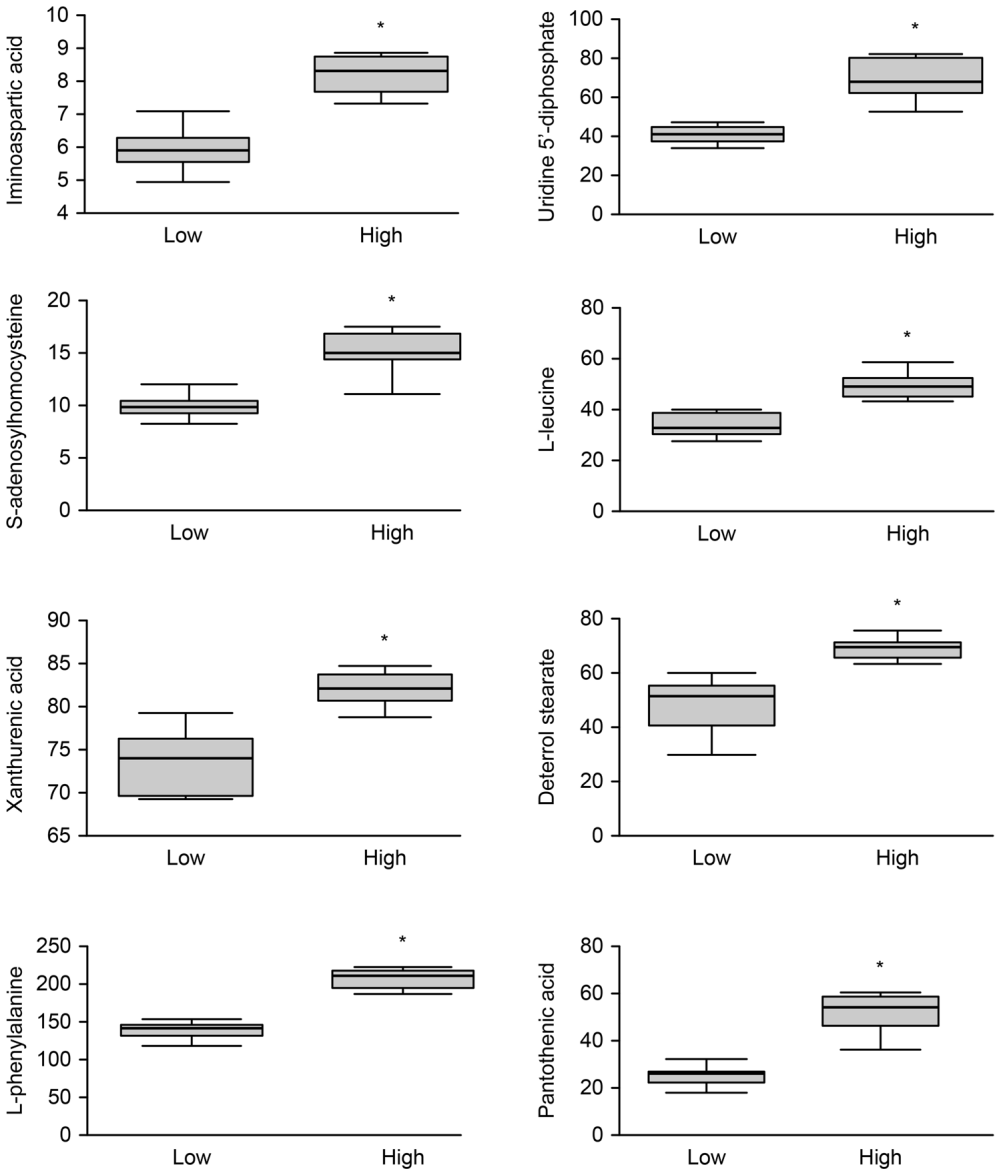
Metabolite profiles obtained from the quantitative analysis of the subjects. The figure was obtained in the same manner as Fig. 2. The differences in iminoaspartic acid, pantothenic acid, L-phenylalanine, L-leucine, deterrol stearate, xanthurenic acid, uridine 5′-diphosphate, and S-adenosylhomocysteine between the LA and HA are displayed. “*” indicates *P* < 0.01.

Twelve mycelial metabolites relevant to fungi were tentatively identified with a mass range tolerance of <0.01 Da through comparisons with the theoretical mass. The detailed peak annotations, assignment and univariate statistical analysis of the metabolites derived from the non-targeted Q-TOF/MS analysis are summarised in Table 13 and Figs 2 and 3. The 12 metabolites were divided into three categories: amino acids, lipids and nucleosides. All amino acids were downregulated at LA compared with the concentrations measured at HA, particularly L-leucine, pantothenic acid and L-phenylalanine, which presented more than a 10-fold decrease. The concentrations of the 2 metabolites classified into the nucleoside category were more than 10-fold higher at HA than at LA. Four of the 6 lipid metabolites decreased at HA. The LysoPC (18:3(9Z,12Z,15Z)) (LPC) and ganolucidic acid C concentrations were 10-fold higher, while LysoPE (18:3(6Z,9Z,12Z)/0:0) (LPE) and (S)-ureidoglycolic acid were 10-fold lower at LA than at HA. The remaining 2 lipid metabolites increased; notably, deterred stearate and xanthurenic acid were reduced by less than 10-fold at LA than the concentrations measured at HA.

**Table 13:** Metabolites showing statistically significant differences between HA and LA.

Name of metabolite	Class	Quantitative ion (m/z)	Retention time (min)	T^α^	*P* ^β^	VIP ^γ^
**Amino acid**						
L-phenylalanine	Amino acid	166.0872	2.655153	−11.76	0.00	1.70
Pantothenic acid	Amino acid	220.1185	2.733965	−12.69	0.00	1.63
L-leucine	Amino acid	132.1027	2.002348	−12.74	0.00	1.55
Iminoaspartic acid	Amino acid	149.0611	2.657355	−7.81	0.00	1.62
**Lipid**						
LPC	Fatty acid esters	518.3328	9.181081	12.84	0.00	1.68
Ganolucidic acid C	Fatty acid	519.3334	9.166005	11.18	0.00	1.64
Deterrol stearate	Prenol lipid	496.4194	10.56405	−8.47	0.00	1.49
LPE	Glycerophospholipid	476.2734	9.506074	7.52	0.00	1.48
(S)-ureidoglycolic acid	Organic acid	152.0587	1.889026	6.70	0.00	1.55
Xanthurenic acid	Organic acid	223.0645	7.879964	−6.97	0.00	1.50
**Nucleosides**						
Uridine 5′-diphosphate	Nucleoside	405.0087	2.33118	−11.19	0.00	1.59
S-adenosylhomocys-teine	Nucleoside	385.1298	4.039654	−12.12	0.00	1.57

^α^Relative concentrations compared with LA: T > 0 indicates upregulated; T < 0 indicates downregulated.

^β^*P* value determined using Student’s t test.

^γ^Correlation coefficient and VIP values were obtained from orthogonal projection to the latent structures discriminant analysis.

## Discussion

In general, the factors affecting microbiological mycelia growth in fermentation include growth conditions and nutrients.

Rotation speed played a significant role in the submerged culture of the fungi. Here, except for the rotation speed of 0 rpm, the effect of other rotation speeds, which ranged from 100 rpm to 180 rpm, on mycelial growth with a capacity of 50 mL in a 250-mL EF were not significant (Table 3). This result was similar to that with a 300 mL capacity in a 500-mL EF, and a rotation speed over 150 rpm increased the mycelial growth of *I*. *farinosa*^25^. Although the results of rotation speed above were similar, there appeared to be no relationship between rotation speed and the capacity for mycelial growth of the fungi. Here, it was clear that the mycelial weight decreased as the capacity increased from 50 to 200 mL in the 250-mL EF. The nutrition from either a 50 or 200 mL capacity in the 250-mL EF was sufficient for mycelial growth over five days (unpublished data). Therefore, the amount of nutrients did not lead to differences in growth. The reason for the differences might be that the rotation speed was too slow for the higher capacities and not favourable for oxygen transport^26,27^. Because 200 mL consumed most of the volume in the 250-mL EF, the 120 rpm rotation speed may not have assisted with oxygen transport. If the rotation speed was higher than 120 rpm, oxygen transport might be increased, and higher mycelial yields might be obtained at higher capacities. Therefore, to improve the mycelial growth of *I*. *farinosa*, high capacities should be paired with high rotation speeds. However, this hypothesis needs further testing.

During the mass industrial production of fungi, the amount of inoculum concentration is an important criterion that not only decides the output of the submerged fungi but also contributes to the final cost. Here, 10% was regarded as the best inoculum concentration, which was same as the concentration reported by Ze *et al*.^28^ for *I*. *farinosa*.

Illumination affects the growth and development of fungi. Arjona *et al*.^29^ reported that the mycelial growth of *Pleurotus ostreatus* (Jacq.) P. Kumm. 1871 was inhibited by white light. Additionally, blue light prevented the mycelial growth of *Monascus ruber* Tiegh. 1884^30^ and *Cordyceps militaris* (L.) Fr. 1818^31^. Illumination prevented the mycelial growth of *O*. *sinensis*^27^. Here, illumination had no significant effect on mycelial growth, and it was found that light did increase the spore yield. This result was different from the report^6^ that the mycelial growth and spore germination of *I*. *farinosa* were inhibited by ultraviolet rays. However, regardless of the effect of illumination on the mycelial growth, it was interesting to further study the connections among light, mycelial growth and spore yield.

Different fungi had different optimal temperatures for mycelial growth^32–34^. Here, 20 °C was the optimal temperature for the mycelial growth of *I*. *farinosa*. This result was the same as that reported by Long *et al*.^6^, but other researchers have reported optimal temperatures of 20–25 °C^22^, 15–25 °C^23^ and 14–18 °C^35^. The different strains of *I*. *farinosa* may be responsible for these different reported results.

There have been many studies on the effects of carbon sources on fungal growth^32,34,36^. Regarding *I*. *farinosa*, Liu *et al*.^24^ compared 16 carbon sources and reported that although D-(−)-arabinose was the optimal carbon source for mycelial growth, there was no significant difference in growth between a medium containing D-(+)-glucose and 10 other carbon sources. Here, based on the results^24^, D-(+)-glucose was selected as the ideal candidate carbon source because of its convenient use and low cost compared with other carbon sources. This selection was similar with the report^37^ that corn flour, sucrose, maltose and glucose were more suitable carbon sources, but it was different from the conclusion^38^ that maltose was the best carbon source for the mycelial growth of *I*. *farinosa*. Different test conditions might be the reason for the difference.

Inorganic nitrogen, amino acids and complex organic nitrogen are typically used to evaluate the best nitrogen source for the mycelial growth of fungi^36^. Among inorganic nitrogen, urea was considered the best nitrogen source for the citric acid yield in *Aspergillus niger* Tiegh. 1867^39^. However, in the present research, based on a prior study^24^, *I*. *farinosa* could not use urea well; otherwise, most of the amino acids were not suitable for the mycelial growth of *I*. *farinosa*, and the mycelial weight was the lowest when L-aspartic acid was the nitrogen source. Among all of the studied nitrogen sources, beef extract, yeast powder and peptone were suitable for the mycelial growth of *I*. *farinosa*. Thus, their prices and convenience were the selection basis for practical application. Although beef extract and yeast powder had similar prices, and both of their prices were slightly higher than that of peptone (Offered by Beijing AOBOX biotechnology Co. Ltd.), yeast powder easily floats in the air during the operation; in addition, both yeast powder and peptone are hygroscopic and easy to harden because of hygroscopicity during storage, which would result in waste during storage or inconvenience during the operation. Therefore, beef extract was selected as the most suitable nitrogen source in the present work. Although yeast powder, peptone and beef extract were determined as suitable nitrogen sources for the mycelial growth of *I*. *farinosa* by different researchers^23,37,38^, the illuminations for their experiments were not shown in the papers, and their range tested was 7 nitrogen sources, which might result in the difference.

The aim of the research on the C/N ratio of *I*. *farinosa* was not only to select the best combination of nutrients to increase mycelial growth but also to take advantage of the nutrition and avoid wasting materials. Here, *I*. *farinosa* grew better when the C/N ratio was 1:1, but the ratio of 7:1 had a similar effect on improving mycelial growth (Table 8). Different fungi have different suitable C/N ratios. For *C*. *sinensis*, the suitable C/N ratio was 12:1, and there were no significant differences among the ratios of 12:1, 18:1, 24:1, 36:1 and 48:1^36^. For *Psathyrella atroumbonata* Pegler 1966, the suitable C/N ratio was 2:3, and the worst C/N ratio was 5:1^40^. Chandra and Purkayastha^41^ reported that the ratios of 1:5, 5:1 and 3:1 were the optimal C/N ratios for the mycelial growth of *Volvariella volvacea* (Bull.) Singer 1951, *Termitomyces eurrhizus* (Berk.) R. Heim 1942 and *Lentinus subnudus* Berk. 1847, respectively. Although the C/N ratio was the same, the method of calculating the C/N ratio was different in different reports. The content of the carbon and nitrogen elements in different carbon and nitrogen sources was applied in the research of *C*. *sinensis*^36^, while the mass ratio method was applied in other research reports^40–42^.

Elements, whether macro-elements or trace elements, played different roles in the physiology of fungi. Here, in a medium without any elements, the mycelial growth of *I*. *farinosa* was the lowest, and the difference was significant. These results demonstrate the importance of the elements for the mycelial growth. In addition, the trace element iron and the macro-element magnesium were ultimately more important than the other elements because the mycelial weights of *I*. *farinosa* in the medium without them were the lowest (with the exception of the no-element controls). However, the results where the mycelial dry weights of complete medium without sodium and complete medium without copper were the highest indicate that sodium and copper were more indispensable than the other elements examined (Tables 9 and 10). Regarding elements, previous studies have indicated that iron is important for *C*. *sinensis*^36^, and calcium is essential for *Irpex lacteus* (Fr.) Fr. 1828^34^ and *Calocybe indica* Purkay. & A. Chandra 1974^41^.

Like many other vitamins, V_B1_ produces a growth response at very low concentrations and typically has a catalytic function in the cell as coenzymes or constituents of coenzymes^43–45^. Therefore, V_B1_ has been widely used in fungal fermentation^46–48^. Because V_B1_ is soluble in water and stable at acidic pH^44^ and because the pH of media would be changed into acid by *I*. *farinosa* during its fermentation^25^, similar with some other entomopathogenic fungi^49,50^, V_B1_ was suitable for the mycelial growth of *I*. *farinosa*, and it has been used in the production of *I*. *farinosa*^25^. Because of the important physiological functions during cellular metabolism and the extensive applications to the fermentation of fungi, V_B1_ was selected as the optimal vitamin to be used in the present study directly based on the effect of the vitamin on the mycelial growth of *I*. *farinosa*^24^.

Studies concerning the effects of altitude on organs, especially in humans^51^, animals^52^, plants^53^ and insects^54^, are numerous; however, few studies have been conducted on the effects of altitude on microorganisms, and these studies have mainly focused on microbial community structures at different altitudes^55,56^. The present study is the first report about the altitude effect on mycelial growth and metabolome. Here, no significant difference in mycelial production was observed between LA and HA, indicating that altitude in the studied range did not influence mycelial growth. The strain used in the study was obtained at HA, and its characteristics, including its differentially regulated metabolites and genes such as hypoxia-inducible factors^57^, likely evolved to adapt to hypobaric and hypoxic HA conditions^58^. These evolved coping strategies of *I*. *farinosa* at HA might be the reason why the mycelial yield at HA was same as that at LA. However, the connections among the 1048 metabolites, the changes in expression of related genes and mycelial growth need to be further researched. Different organisms have different methods of adapting to HA. For example, pregnant women adapt to HA by increasing their ability to maintain maternal oxygenation^59^; insects at HA have reduced their oxygen requirements to adapt to hypoxia by having smaller body sizes and reduced capabilities of wings and flight^54^; additionally, bar-headed geese have evolved a muscular phenotype for extreme HA flight^60^.

The results for all mycelial samples that showed the apparent separation between the LA and HA mycelial samples based on the regulated metabolite deeply indicate that although the mycelial production was the same, the metabolome of mycelia was significantly regulated by altitude. The significantly regulated metabolites may be biomarkers for the effects of altitude on *I*. *farinosa*. However, this regulation of metabolites suggested that the preservation of fungal quality is a significant and difficult challenge for medicine. Under certain environmental stressors, such as salinity, temperature, and hypoxia^61^, the concentrations of specific metabolites in organisms may change significantly, with greater stress resulting in more substantial changes. Because each metabolite interacts with other metabolites in the metabolic network, the reaction of one metabolite to stress must result in the regulation of the other related metabolites, perhaps including some substances that are harmful to humans. Therefore, these changes will increase the instability of quality and result in a safety risk of the application of the fungus for a medicine. A similar potential change might be observed in Chinese *Cordyceps* which was recorded by the Chinese Pharmacopoeia^62^. Chinese *Cordyceps* can be found at an altitude from 2200 to 5000 metres in the Tibetan Plateau^63^. According to the present results, changes in altitude might result in significant changes in the metabolome of Chinese *Cordyceps*. However, no restrictions regarding altitude are noted for Chinese *Cordyceps* in the Chinese Pharmacopoeia. Therefore, to preserve fungal quality, establishing standard culturing methods is necessary, and once the methods concerning altitude are resolved, the safety of fungi produced at different altitudes must be further investigated systematically by analysing the metabolome.

Notably, regarding the production of specific metabolites from mycelia, the altitude that is suitable for promoting the upregulation of beneficial metabolites is optimal. Hence, based on the observed changes in metabolites with altitude, different changes in *I*. *farinosa* may require different suitable altitudes for fermentation. This study presented a new way to select a better method for producing more beneficial materials. Among the 12 identified metabolites, phenylalanine is an essential amino acid widely used in food and medicine (http://www.dcnutrition.com). Most studies have focused on high-production methods of L-phenylalanine, including chemical synthesis, enzymatic methods and fermentation^64^, among which fermentation is optimal because of its low cost, minimal pollution and high product purity^65^. Here, the results showed that the expression of L-phenylalanine increased significantly at HA (Fig. 3), demonstrating an effective strategy for increasing L-phenylalanine production. However, through the non-targeted metabolome pattern analysis^66^, some medical compounds, such as paecilosetin or militarinones B, were not found among the 1048 expressing compounds. This result just shows that the difference in their expressions at HA and LA were not significant, deeply indicating that it was not optimal to regulate their yield per unit mycelia by changing the altitude among the studied range.

In summary, similar to variables such as temperature, nitrogen sources and many other factors, altitude is one of the important cultural conditions of *I*. *farinosa*, and the metabolome of the fungus is regulated by altitude. The effect of altitude on the fungus makes it possible to produce more beneficial metabolites at suitable altitudes, which presents a new way for producing more beneficial metabolites according to their regulation at different altitudes. However, the effect of altitude is a double-edged sword, and some metabolites that are harmful to humans may be produced at the same time, which indicates the necessity of establishing standard culturing methods related to altitude to preserve fungal quality. In addition, the results of the study make it possible to use a fermenter to meet the demands of large-scale mycelial production, which is an ongoing project in this laboratory.

## Materials and Methods

### Fungal strain

Strain number 48^24^ was used in this study. The strain was originally isolated from the larvae of *T*. *gonggaensis*, which were raised at an altitude of 3800 m in an artificial cultivation base.

### Inoculum preparation

First, the strain was incubated in a Petri dish on potato dextrose agar (PDA) at 20 °C. Five days later, a 5-mm agar disc with mycelia of *I*. *farinosa* was punched with a sterilized cutter from the culture and transferred to a fresh Petri dish with PDA for solid culture and to a 500-mL EF containing 100 mL potato dextrose broth (PDB) for liquid culture, which was rotated at 150 rpm, had a photoperiod of 12 h light/12 h darkness and 20 °C for 6 d (used as the seed culture in the study on culture condition) or 5 d (used as the seed culture in the study on nutrition effect, orthogonal experiment and fermentation at different altitudes).

### Culture conditions

#### Basal medium and culture conditions for mycelial growth

All optimal culture conditions for mycelial growth were determined by examining one factor at a time. PDA was used as the basal medium for the temperature research (20 mL of PDA was added to a 9-cm Petri dish), and PDB was used as the basal medium for the other culture condition experiments, including the verification test on temperature. Unless otherwise specified, the following conditions were performed: the medium was initially a pH of 6^25^ [Adjusted with 1.0 N HCl or 1.0 N NaOH before sterilization and determined using a Sartorius Basic pH meter PB-10 (Germany). The following pH values were adjusted the same way], and 50 mL medium in a 250-mL flask was autoclaved at 121 °C for 30 min. All cultures were inoculated with 5% by volume seed culture and agitated at 120 rpm in 12 h darkness/12 h light with 50–55 lux at 20 °C for 5 d.

The mycelial growth rate for the temperature experiments on PDA was determined by colonial diameter, and the mycelia for the other treatments were harvested after 5 d by centrifugation for 10 min at 8000 × *g* to separate the sample from the liquid medium. The mycelial pellets were washed three times with distilled water and dried to a constant dry weight at 60 °C.

#### Effect of capacity

There were 5 treatments for capacity. In one treatment, 100 mL of PDB was added to a 500-mL EF. In the other treatments, 50, 100, 150 and 200 mL of media were added to respective 250-mL EFs.

#### Effect of inoculum concentration

Five treatments were created that focused on the inoculum concentration, and these included 2, 5, 10, 15 and 20% seed cultures (volume ratio).

#### Effect of illumination

The treatments for the illumination experiment included the following photoperiods: 24 h of darkness, 12 h of darkness/12 h of light and 24 h of light. For the treatment with 24 h of darkness, a 250-mL EF was wrapped with silver paper. For the treatment with 12 h of darkness/12 h of light, the samples were kept in a light room during the light portion of the cycle, and the light intensity was 300 lux, which was same as that of the treatment with 24 h of light.

#### Effect of rotation speed

There were 5 treatments used for the rotation speed experiments, and these included 0, 100, 120, 150 and 180 rpm.

#### Effect of temperature

The temperatures ranged from 0 to 35 °C with a 5 °C temperature interval between each subsequent treatment. The colony diameter was measured after 5 d to determine the growth rate. Based on the results, the verification experiments at 15, 20 and 25 °C were conducted in liquid fermentation for 5 d.

### Nutrition effect

#### Basal medium and culture conditions for mycelial growth

All nutrition requirements for mycelial growth were determined by examining one individual factor at a time. In the following experiments, glucose was autoclaved separately and V_B1_ was sterilized by filtration through a 0.22-μm-pore-size filter unit, and then both were added to the cooled autoclaved medium. The basal medium and the culture condition are shown in Table 14. The effects of different carbon and nitrogen sources were tested with a pH of 6.5 by inoculating 2.5 mL of mycelial seed culture and incubating the cultures on a rotary shaker at 150 rpm at 20 °C for 5 d, which was in accordance with the report by Liu *et al*.^24^. Unless otherwise specified, the media for the other nutrition experiments were tested with a pH of 6^25^ (same as the pH of the culture conditions part) by inoculating with 5 mL of mycelial seed culture and agitated under 12 h light (the light intensity was 50–55 lux)/12 h darkness at 120 rpm and 20 °C for 5 d.

**Table 14:** Basal medium and culture conditions for the mycelial growth of *Isaria farinosa* during some experiments.

Experiment	Basal medium	Culture condition
Carbon and nitrogen source	0.5 g L^−1^ of MgSO_4_ 7H_2_O, 0.5 g L^−1^ of KH_2_PO_4_, 0.65 g L^−1^ of Na_2_HPO_4_, 0.5 g L^−1^ of KCl and 0.5 mg L^−1^ V_B1_. (11 g L^−1^ of glucose and 2 g L^−1^ of NaNO_3_).	50 mL/250 mL, 5% inoculum, 150 rpm, 12 h light (The light intensity was 50–55 lux)/12 h darkness, initial pH = 6.5 and 20 °C.
C/N ratio	0.5 g L^−1^ of MgSO_4_ 7H_2_O, 0.5 g L^−1^ of KH_2_PO_4_, 0.65 g L^−1^ of Na_2_HPO_4_, 0.5 g L^−1^ of KCl and 0.5 mg L^−1^ of V_B1_. (D-(+)-glucose and beef extract).	50 mL/250 mL, 10% inoculum, 120 rpm, 12 h light (The light intensity was 50–55 lux)/12 h darkness, initial pH = 6 and 20 °C.
Macro and trace element*	11 g L^−1^ of D-(+)-glucose, 0.7050 g L^−1^ of CO(NH_2_)_2_ and 0.5 mg L^−1^ of V_B1_.	Same as that of C/N ratio except the natural pH value
Optimal combination by using orthogonal matrix method	14.20 g L^−1^ of D-(+)-glucose, 4.00 g L^−1^ of beef extract, 1.50 mg L^−1^ of V_B1_, 0.5 g L^−1^ of MgSO_4_·7H_2_O, 0.0498 g L^−1^ of FeSO_4_·7H_2_O, 0.5 g L^−1^ of KH_2_PO_4_, 0.5 g L^−1^ of KCl and 0.65 g L^−1^ of Na_2_HPO_4_.	Same as that of C/N ratio.
Fermentation at different altitudes	Same as that for optimal combination by using orthogonal matrix method.	Same as that of C/N ratio except 24 h darkness.

*The basal medium excluding the test factor (s).

The mycelia were tested using the drying method described above.

#### Effect of carbon source

Sixteen carbon sources were tested (Table 6). Based on the study of carbon sources^24^, D-(−)-arabinose was excluded because of its lower solubility, and cellulose was studied as a carbon source in the study. A medium that was free of any carbon source served as a control.

#### Effect of nitrogen source

Nineteen tested nitrogen sources are shown in Table 7. Based on the study of nitrogen sources^24^, L-tyrosine was excluded because of its lower solubility, and sodium nitrate and complex organic nitrogen were studied as the nitrogen sources in the study. The medium lacking a nitrogen source was control 1, and the medium lacking both a nitrogen source and a carbon source was control 2.

#### Effect of C/N ratio

According to the results of both the carbon and nitrogen sources, D-(+)-glucose and beef extract were used to test the C/N ratios. With the same total organic matter, eight liquid media with different C/N ratios were prepared^67^, as shown in Table 8.

#### Effect of macro-element

The tested macro-elements included calcium, magnesium, potassium and sodium. The complete medium for the macro-element tests contained 0.5 g L^−1^ of CaCl_2_·2H_2_O, 0.5 g L^−1^ of KH_2_PO_4_, 0.5 g L^−1^ of MgSO_4_·7H_2_O, 0.5 g L^−1^ of Na_2_HPO_4_, 11 g L^−1^ of D-(+)-glucose, 0.7050 g L^−1^ of CO(NH_2_)_2_ and 0.5 mg L^−1^ of V_B1_ in distilled water. The necessity of each macro-element was determined by excluding one element from the complete medium at a time. A complete medium with all tested macro-elements was used as control 1, and a basal medium lacking all tested macro-elements was used as control 2.

#### Effect of trace elements

The tested trace elements included copper, iron, manganese and zinc. The experiment used the same approach as the macro-element experiment, but the concentrations of the trace elements were 0.0393 g L^−1^ of CuSO_4_·5H_2_O, 0.0498 g L^−1^ of FeSO_4_·7H_2_O, 0.0308 g L^−1^ of MnSO_4_·H_2_O, 0.0439 g L^−1^ of ZnSO_4_·7H_2_O, 11 g L^−1^ of D-(+)-glucose, 0.7050 g L^−1^ of CO(NH_2_)_2_ and 0.5 mg L^−1^ of V_B1_ in distilled water. A complete medium with all tested trace elements was used as control 1, and a basal medium lacking all tested trace elements was used as control 2.

### Orthogonal matrix method

Nutrition is one of the most important factors affecting the mycelial growth of fungi. Based on the results obtained by testing one factor at a time, D-(+)-glucose, beef extract and V_B1_^24^ were selected as the carbon, nitrogen and vitamin sources, respectively, used to study the optimal combination by using the orthogonal L_8_(2^7^) matrix method. The levels of these components in the culture medium are shown in Table 15, and the other nutritional and culture conditions are shown in Table 14.

**Table 15:** L_8_(2^7^) orthogonal design for optimisation of the culture medium.

Level	D-(+)-glucose (g L^−1^)	Beef extract (g L^−1^)	V_B1_ (mg L^−1^)
Level 1	8.50	2.00	0.50
Level 2	14.20	4.00	1.50

### Fermentation metabolome at different altitudes

#### Sample preparation

Based on the optimal culture nutrition and conditions (Table 14), fermentation was simultaneously conducted at HA and LA. All experiments were performed ten times. The mycelial pellets were washed three times with distilled water and vacuum freeze-dried to a constant weight. Finally, all samples were frozen at –80 °C until further use.

Based on the results of previous studies and another report^68^, the mycelial aliquots (50 mg) were thawed at 4 °C and ultrasound-extracted for 1 h with the addition of 10 mL of ice-cold methanol/water (4:3 vol/vol). After centrifugation for 10 min (10000 × *g*, 4 °C), 1 mL of each supernatant was transferred to a Q-TOF/MS autosampler vial. To confirm the reproducibility of the Q-TOF/MS system, quality control samples were prepared in parallel by mixing 10 μL of supernatant for each sample.

#### Instrument conditions

The experiments were performed using the Triple TOF™ 5600 system (AB SCIEX, Framingham, MA, USA) fitted with a UPLC 20 A system (Shimadzu, Japan). A Kinetex XB-C18 column (2.1 mm × 100 mm, 2.6 μm, 100 Å, Phenomenex) was used with the binary gradient method. The mobile phase was 0.1% formic acid in ultra-pure water (A)-acetonitrile (B). A flow rate of 300 μL/min was used, and the injection volume was 2 μL. The gradient programme used was 10% B at 1 min, 80% B for 1 min to 5 min, 80% B at 7 min, and 10% B at 7.01 min, for a total run time of 10 min.

#### MS/MS conditions

The AB SCIEX Triple TOF 5600 LC/MS/MS system with a Duo-spray source using an ESI probe for analysis and an atmospheric-pressure chemical ionisation probe for calibration was used to acquire the original data. A semi-targeted generic implicit differential-algebraic method was applied to develop a list of expected metabolites. PeakView^TM^ was used to detect the raw data after analysis by the LC/MS/MS system, and the data were further processed and analysed with MarkView^TM^ software.

### Data processing and statistical analysis

The raw data detected by PeakView^TM^ (version 1.2.1) software from the LC/TOF-MS system were processed with baseline corrections, scaling and peak alignment using MarkView v1.2.1 (ABSciex). Next, several aligned peaks from the raw data were extracted to create ordered data matrices using a software package. The parameters of the software were set as follows: a minimum RT of 0.9 min, a maximum RT of 30 min, a subtraction offset of 10 scans, a noise threshold of 5 and a maximum number of peaks of 2000. After this procedure, data spreadsheet files on the peak intensities were generated and exported to Microsoft Office Excel (Microsoft Corp., Redmond, WA, version 2010), and each peak required the identification of the exact m/z and RT. The number of peak intensities was further handled for pattern analysis using PCA and PLS-DA. With the exception of effects of altitude on mycelial growth and their metabolomes, all experiments were performed in triplicate. The data were analysed with one-way ANOVA. Significance was determined by Duncan’s multiple range tests or by Student’s t-test at *P* = 0.05 using SPSS 17.0 (SPSS INC., Chicago, IL, USA).

## Electronic supplementary material

Tables S1–3

Dataset S1

Dataset S2

## Data Availability

All data generated or analysed during this study are included in this published article (and its Supplementary Information files).

## References

[1] Zimmermann G (2008). The entomopathogenic fungi *Isaria farinosa* (formerly *Paecilomyces farinosus*) and the *Isaria fumosorosea* species complex (formerly *Paecilomyces fumosoroseus*): biology, ecology and use in biological control. Biocontrol. Sci. Techn..

[2] Zeng W, Chen SJ (2001). Studies on *Paecilomyces muscardine* of *Cordyceps sinensis* host insect. *Chin*. J. Chin. Mater. Med..

[3] Lopes RD (2017). The potential of *Isaria* spp. as a bioinsecticide for the biological control of *Nasutitermes corniger*. Biocontrol Sci. Techn..

[4] Lu YH (2018). Symptoms, infection and histopathology of *Hepialus* sp. larvae parasitized by *Isaria farinosa*. Mycosystema.

[5] He YC, Lu ZH, Chen SJ (2017). Evaluation of germicidal efficacy and toxicity of polyhexamethylene biguandine disinfection solution against *Hepialus* larva pathogen *Isaria farinosa*. Mycosystema.

[6] Long YL (2017). Biological characteristics and miticide activity of *Paecilomyces farinosus* to *Panonychus citri*. J. Hunan Agr. Univ. (Nat Sci).

[7] Linke D (2017). Cold generation of smoke flavour by the first phenolic acid decarboxylase from a filamentous ascomycete — *Isaria farinosa*. Fungal Biol-UK.

[8] Kozłowska E (2018). Biotransformation of steroids by entomopathogenic strains of *Isaria farinosa*. Microb. Cell Fact..

[9] Liu F (2016). Progress on molecular biology of *Isaria farinosa*, pathogen of host of *Ophiocordyceps sinensis* during the artificial culture. *Chin*. J. Chin. Mater. Med..

[10] He XH (2017). Cloning and analysis of encoding cDNA sequence of sublitisin-like protease gene from *Isaria farinosa*. Guangdong Agr Sci..

[11] Lu ZH, Wu XL, Liu F (2013). Advance on the active and pesticide effection of the ingredient of *Isaria farinosa*. *Chin*. Pharm. J..

[12] Cheng Y (2004). Farinosones A-C, neurotrophic alkaloidal metabolites from the entomogenous deuteromycete *Paecilomyces farinosus*. J. Nat. Prod..

[13] Wang P, Jiang X, Jiang Y, Hu X, Hwang H (2008). Optimization of fermentation medium and conditions for mycelial growth and water-soluble exo-polysaccharides production by *Isaria farinosa* B05. Prep. Biochem. Biotechnol..

[14] Zhang YG, Liu SC, Liu HW, Liu XZ, Che YS (2009). Cycloaspeptides F and G, cyclic pentapeptides from a *Cordyceps*-colonizing isolate of *Isaria farinosa*. J. Nat. Prod..

[15] Velmurugan P (2010). Water-soluble red pigments from *Isaria farinosa* and structural characterization of the main colored component. J. Basic Microbiol..

[16] Lang G, Blunt JW, Cummings NJ, Cole ALJ, Munro MHG (2005). Paecilosetin, a new bioactive fungal metabolite from a New Zealand isolate of *Paecilmyces farinosus*. J. Nat. Prod..

[17] Ma C (2011). N-hydroxypyridones, phenylhydrazones, and a quinazolinone from *Isaria farinosa*. J. Nat. Prod..

[18] Jiang YH, Jiang XL, Wang P, Hu XK (2005). *In vitro* antioxidant activities of water-soluble polysaccharides extracted from *Isaria farinosa* B05. J. Food Biochem..

[19] Lu HE (2010). Hypoglycaemic effects of fermented mycelium of *Paecilomyces farinosus* (G30801) on high-fat rats with streptozotocin-induced diabetes. Indian J. Med. Res..

[20] Jiang YH (2008). The antitumor and antioxidative activities of polysaccharides isolated from *Isaria farinosa* B05. Microbiol. Res..

[21] Mascarin GM, Alves SB, Lopes RB (2010). Culture media selection for mass production of *Isaria fumosorosea* and *Isaria farinosa*. Braz. Arch. Biol. Technol..

[22] An JM (2003). Study on the culture conditions of an entomology fungi, *Paecilomyces farinosus*. J. Shanxi Teach. Univ. (Nat. Sci. Edit).

[23] Zhao LF, Wang HL (2008). Effects of several ecological factors on growth and spore production of *Paecilomyces farinosus*. *J*. *West Chin*. For. Sci..

[24] Liu F (2016). Nutritional effects on the mycelial growth and enzymatic activity of *Isaria farinosa*, and *Hepialus* larvae growth. J. Appl. Microbiol..

[25] Yang B, Li TS, Liu CY, Yao JP, Wu TL (2005). Studies on the impact of rotate speed of rocking bed, initial inoculums and initial pH value on mycelial bioass of *Paecilomyces farinosu*s. J. Yunnan Univ. (Nat. Sci.).

[26] Li R, Li J, Zhao WQ, Li KF (2000). Study on reaeration laws of turbulent water body. Acta Scien. Circum..

[27] Dong CH, Yao YJ (2011). On the reliability of fungal materials used in studies on *Ophiocordyceps sinensis*. J. Ind. Microbiol. Biot..

[28] Ze SZ, Zhou N, Liu HP, Chen P, Li HR (2006). Production process of *Paecilomyces farinosus*, an entomopathgenic fungus. *J*. *West Chin*. For. Sci..

[29] Arjona D, Aragon C, Aguilera JA, Ramirez L, Pisabarro AG (2009). Reproducible and controllable light induction of *in vitro* fruiting of the white-rot basidiomycete *Pleurotus ostreatus*. Mycol. Res..

[30] Zhang XW (2017). Effects and mechanism of blue light on *Monascus* in liquid fermentation. Molecules.

[31] Yang T, Dong CH (2014). Photo morphogenesis and photo response of the blue-light receptor gene *Cmwc-1* in different strains of *Cordyceps militaris*. FEMS Microbiol. Lett..

[32] Bae JT, Sinha J, Park JP, Song CH, Yun JW (2000). Optimization of submerged culture conditions for exo-biopolymer production by *Paecilomyces japonica*. J. Microbiol. Biotechn..

[33] Park JP, Kim SW, Hwang HJ, Yun JW (2001). Optimization of submerged culture conditions for the mycelial growth and exo-biopolymer production by *Cordyceps militaris*. Lett. Appl. Microbiol..

[34] Dong XM, Song XH, Dong CH (2017). Nutritional requirements for mycelial growth of milk-white toothed mushroom, *Irpex lacteus* (Agaricomycetes), in submerged culture. Int. J. Med. Mushrooms.

[35] Feng YY (2006). The optimal temperature in production of *Paecilomyces farinosus*. *For*. *Invent*. Plann..

[36] Dong CH, Yao YJ (2005). Nutritional requirements of mycelial growth of *Cordyceps sinensis* in submerged culture. J. Appl. Microbiol..

[37] Yang B, Liu CY, Yao JP, Wu TL (2005). The influence of carbonaceous compounds, nitrogenous substances and inorganic matter on the mycelia biomass of *Paecilomyces farinosus* cultured in liquid media. J. Southwest For. Coll..

[38] Wang SY, Fan CY, Tian RM, Shi YQ, Lu Q (2002). Growth of *Paecilomyces farinosus* under different condition. Inner Mongolia For. Sci. Tech..

[39] Ghosh P (1998). & Bank, A.K. Effect of chemical nutrients on aconitase activity during citric acid fermentation by a mutant strain of *Aspergillus niger*. Acta Microbiol. Pol..

[40] Ghosh P, Fasidi IO (2001). Effect of carbon, nitrogen and mineral sources on growth of *Psathyrella atroumbonata* (Pegler), a Nigerian edible mushroom. Food Chem..

[41] Chandra A, Purkayastha RP (1977). Physiological studies on Indian edible mushrooms. Trans. Brit. Mycol. Soc..

[42] Kim SW (2002). Influence of nutritional conditions on the mycelial growth and exopolysaccharide production in *Paecilomyces sinclairii*. Lett. Appl. Microbiol..

[43] Garraway, M. O. & Evans, R. C. *Fungal nutrition and physiology*. 401 pp (New York: Wiley, 1984).

[44] Wang, J. Y., Zhu, S. G. & Xu, C. F. *Biochemistry* (Vol. 1), 3rd edition. 441–443 pp. (Beijing: Higher Education Press, 2002).

[45] Lohmann K, Schuster P (1937). Untersuchungen über die Cocarboxylase. Biochem. Z..

[46] Liu S (2017). Study on the synthesis of active components from *Ophiocordyceps Xuefengensis* by liquid fermentation. Chin. Pharm..

[47] Ruan Y (2014). The influence of vitamin B_1_, B_6_ and growth hormone 2, 4-D on the production of cordycepin in the liquid fermentation of *Cordyceps militaris*. Mycosystema.

[48] Liu XZ (2017). Effects of medium nutrition on growth of *Grifola frondosa* mycelium and production of extracellular polysaccharides. *Chin*. Agr. Sci. Bull..

[49] St Leger RJ, Nelson JO, Screen SE (1999). The entomopathogenic fungus *Metarhizium anisopliae* alters ambient pH, allowing extracellular protease production and activity. Microbiology.

[50] Asaff A, Cerda-García-Rojas CM, Viniegra-González G, Torre MDL (2006). Carbon distribution and redirection of metabolism in *Paecilomyces fumosoroseus* during solid-state and liquid fermentations. Process Biochem..

[51] Nocturnal Oxygen Therapy Trial Group. Continuous or nocturnal oxygen therapy in hypoxemic chronic obstructive lung disease: a clinicaltrial. *Ann Intern Med*. **93**, 391–398 (1980). 10.7326/0003-4819-93-3-391 6776858

[52] Koundal S, Gandhi S, Kaur T, Mazumder A, Khushu S (2015). “Omics” of high altitude biology: A urinary metabolomics biomarker study of rats under hypobaric hypoxia. OMICS J. Integr. Biol..

[53] Ling TX (2017). Effect of the altitude on the normal chemical composition, content and coordination of the flue-cured tobancco in Wushan. Jiangsu Agr. Sci..

[54] Mani, M. S. *Ecology and Biogeography of High Altitude Insects*, Series entomologica Vol. 4. 527 pp. (The Hague: Dr W. Junk N. V. Publishers, Belinfante, NV, 1968).

[55] Ren CJ (2018). Differential responses of soil microbial biomass, diversity, and compositions to altitudinal gradients depend on plant and soil characteristics. Sci. Total Environ..

[56] Tian JQ (2017). Patterns and drivers of fungal diversity along an altitudinal gradient on Mont Gongga, *Chin*. J. Soil Sediment.

[57] Storz JF (2010). Genes for high altitudes. Science.

[58] Hoback WW, Stanley DW (2001). Insects in hypoxia. J. Insect Physiol..

[59] McAuliffe F, Kametas N, Krampl E, Ernsting J, Nicolaides K (2001). Blood gases in pregnancy at sea level and at high altitude. Bjog.

[60] Scott G. R., Egginton S., Richards J. G., Milsom W. K. (2009). Evolution of muscle phenotype for extreme high altitude flight in the bar-headed goose. Proceedings of the Royal Society B: Biological Sciences.

[61] Garreta-Lara E, Campos B, Barata C, Lacorte S, Tauler R (2018). Combined effects of salinity, temperature and hypoxia on *Daphnia magna* metabolism. Sci. Total Environ..

[62] Pharmacopoeia Commission of P.R.C. *Pharmacopoeia of the People’s Republic of China* 115 pp. (China Medical Science Press, 2015).

[63] Liu, F., Wu, X. L., Yin, D. H., Chen, S. J. & Zeng, W. Study on the biology characters of host insect of *Ophiocordycepes sinensis*. *Chongqing**J*. *Res*. *Chin*. *Drug Herb*. 46–52 (2015).

[64] Chem SJ (2002). & Consulting Limited Company & Editorial Department of Fine and Specialty Chemicals. The current situation and development prospect of L-phenylalanine. Fine Spec. Chem..

[65] Yue H, Yuan Q, Wang W (2007). Enhancement of L-phenylalanine production by [beta]-cyclodextrin. J. Food Eng..

[66] Issaq, H.J. & Veenstra, T.D. (Hu, Q. Y. & Hou, H. W. translation). *Proteomic and Metabolomic Approaches to Biomarker Discovery*. 433 pp (Beijing: Science Press, 2016).

[67] Wu J, Cheung PCK, Wong K, Huang N (2004). Studies on submerged fermentation of *Pleurotus tuber-regium* (Fr.) Singer. Part 2: Effect of carbon-to-nitrogen ratio of the culture medium on the content and composition of the mycelia dietary fibre. Food Chem..

[68] Luo FF (2014). Identification of spore germination and virulence related biomarkers from *Beauveria bassiana* using an LC-MS-based metabolomic technique. Acta Microbiol. Sin..

