# Supplementary material for: Culture conditions and nutrition requirements for the mycelial growth of Isaria farinosa (Hypocreales: Cordycipitaceae) and the altitude effect on its growth and metabolome
Source: Sci Rep. 2018 Oct 23;8:15623. doi: 10.1038/s41598-018-33965-z (PMC6199337; doi:10.1038/s41598-018-33965-z)
Supplement: Supplementary file 1 — Tables S1–3 [file 41598_2018_33965_MOESM1_ESM.docx]

**Culture conditions and nutrition requirements for the mycelial growth of *Isaria farinosa* and the altitude effect on its growth and metabolome**

**Fei Liu, Mei-Chun Xiang, Yan-Lei Guo, Xiao Li Wu, Guang Xin Lu, Yong Yang, Xing Zhong Liu, Shijiang Chen, Guo Zhen Zhang & Wang Peng Shi**

**Table S1. Growth effects of combinations of D-(+)-glucose and V_B1_ as indicated by mycelial dry weight (g L^-1^).**

|  | **V_B1_ 1** | **V_B1_ 2** |
| --- | --- | --- |
| D-(+)-glucose 1 | 4.44 | 4.67 |
| D-(+)-glucose 2 | 4.70 | 4.56 |

**Table S2. Growth effects of combinations of beef extract and V_B1_ as indicated by mycelial dry weight (g L^-1^).**

|  | **V_B1_ 1** | **V_B1_ 2** |
| --- | --- | --- |
| Beef extract 1 | 4.00 | 3.99 |
| Beef extract 2 | 5.14 | 5.24 |

**Table S3. Growth effects of combinations of D-(+)-glucose and beef extract as indicated by mycelial dry weight (g L^-1^).**

|  | **Beef extract 1** | **Beef extract 2** |
| --- | --- | --- |
| D-(+)-glucose 1 | 3.94 | 5.17 |
| D-(+)-glucose 2 | 4.05 | 5.21 |
